# Prognostic significance of *CTNNB1* mutation in hepatocellular carcinoma: a systematic review and meta-analysis

**DOI:** 10.18632/aging.205047

**Published:** 2023-09-20

**Authors:** Genlin Lu, Jian Lin, Guoqiang Song, Min Chen

**Affiliations:** 1 Department of General Surgery, Longyou People’s Hospital Affiliated with Sir Run Run Shaw Hospital, Zhejiang University School of Medicine, Quzhou 324400, China; 2 Department of Pulmonary, Department of Cancer Center, Changxing Hospital of Traditional Chinese Medicine, Huzhou 313100, China

**Keywords:** hepatocellular carcinoma, prognosis, CTNNB1, mutation, survival

## Abstract

Backgrounds: Hepatocellular Carcinoma (HCC) is one of the most common malignant cancers in humans and has a high fatality rate. In recent years, researchers have verified that the Wnt/β-catenin signaling pathway affects the clinicopathological features and prognosis of patients with HCC. Although many studies have investigated the relationship between Wnt/β-catenin signaling pathway and HCC, the prognostic value of β-catenin in HCC remains inconclusive. *CTNNB1* (Catenin Beta-1) is an important factor in the Wnt/β-catenin signaling pathway. However, no consensus has been reached on the clinical and prognostic significance of *CTNNB1* mutations in HCCs.

Methods: Eligible studies and relevant data were obtained from PubMed, Web of Science, Elsevier, Cochrane Library, Ovid, and Embase databases. The correlation between *CTNNB1* mutations and clinical/prognosis of patients were evaluated. A fixed- or random-effects model was used to calculate pooled odds ratios (OR) and 95% confidence intervals (CI).

Results: Seventeen studies matched the selection criteria, and 1828 patients were included. This meta-analysis demonstrated that patients with HCC with *CTNNB1* mutations had favorable clinicopathological features and survival. The combined ORs of 1-, 3- and 5-year overall survival were0.52 (n = 6 studies, 95% CI: 0.34–0.81, Z = 2.89, *P* =0.004, 0.28 (n =6 studies, 95% CI: 0.18–0.42, Z = 6.03, *P*<0.00001), -0.22 (n = 6 studies, 95% CI: 0.37–0.06, Z = 2.78, *P* = 0.005), respectively. Additionally, *CTNNB1* mutation might be significantly associated with differentiation (OR = 0.54, 95% CI:0.36–0.81, Z = 2.98, *P* = 0.003), TMN stages (Tumor, Node, Metastasis staging classification) (OR = -0.25, 95% CI:-0.33–-0.18, Z = 6.60, *P*<0.00001), liver cirrhosis (OR = 0.21, 95% CI:0.11–0.39, Z = 4.94, *P*< = 0.00001), and HBV (Hepatitis B Virus) infection (OR = 0.44, 95% CI:0.31–0.64, Z = 4.37, *P*<0.0001), but not with tumor size, metastasis, vascular invasion, and HCV infection.

Conclusions: *CTNNB1* mutation can serve as an indicator of favorable prognosis as well as a novel target for treatment in HCC.

## INTRODUCTION

Hepatocellular carcinoma (HCC) causes nearly half a million deaths annually worldwide. Owing to the increasing incidence of hepatitis B virus (HBV) infection, HCC has become a fast-growing cancer in Asian countries, especially in China [1]. The incidence of HCC is expected to increase in the future. However, few therapies can improve the prognosis of patients with HCC [2]. Surgery is the primary curative treatment for HCC. However, no more than 50% of patients survive longer than five years after surgery, even when diagnosed and operated at an early stage [3]. Recently, molecular targeted therapy has brought a glimmer of hope for the treatment of HCC [4]. Owing to its favorable overall survival, the FDA approved the multi-kinase inhibitor sorafenib for the treatment of advanced HCC [5, 6]. As a targeted anticancer molecule, sorafenib has demonstrated only partial efficacy in advanced HCC. Therefore, it is mandatory to better understand the genes and signaling pathways involved in the tumorigenesis and progression of HCC and to identify more effective druggable targets for improving HCC management.

Over the past few years, Wnt pathway activation in HCC has been reported in several studies [7, 8]. This pathway has been indicated to play an important role in the clinicopathological features and prognosis of HCC [9]. According to a recent report, *CTNNB1* is one of the most frequently mutated genes in HCC [10]. Mutation of *CTNNB1*, which is the key downstream effector of the pathway, appears to be the main cause of activation of the Wnt pathway [11]. β-catenin, a protein expressed by *CTNNB1*, integrates the intercellular E-cadherin–catenin adhesion system, the disruption of which has been observed in HCC [12]. β-catenin is normally located in the cytomembrane and is directly connected to E-cadherin, which in turn forms an adhesion complex. This adhesion complex, which can be degraded by phosphorylation or ubiquitination, can regulate cell-cell adhesion and maintain tissue architecture and function. In HCC, unbound β-catenin translocates to the nucleus and regulates the transcription of target genes relevant to cell proliferation and cell cycle progression. β-catenin accumulation in the cytoplasm and/or nucleus is thought to be closely associated with poor prognosis and deep invasion in HCC patients, independent of tumor stage [13]. Recently, mutations of *CTNNB1* have been detected in human HCC, but the clinical implications of the *CTNNB1* mutation are still unclear. Our meta-analysis showed that mutant *CTNNB1* was associated with favorable clinical outcomes and survival in patients with HCC.

## MATERIALS AND METHODS

### Study selection

PubMed, Web of Science, Elsevier, Cochrane Library, Ovid, and Embase databases were searched for articles published until January 20, 2023. The terms used in the search were “*CTNNB1* or beta-catenin, or β-catenin” and “prognostic or prognosis or survival” and “hepatocellular carcinoma or HCC or liver cancer or liver tumor or hepatic cancer or liver tumor or liver neoplasms”. The reference lists of all retrieved articles were manually searched. Only studies published in English were included. Two reviewers (GLL and GQS) completed the systematic literature search and extracted the following parameters from each study: study population characteristics, number of participants, sex ratio, first author, and year of publication.

### Criteria for inclusion and exclusion

Inclusion criterion:

1. Patients with HCC were diagnosed by pathology;
2. Information about *CTNNB1* mutation, OS (Overall Survival), and other clinicopathological features were provided;
3. The *CTNNB1* mutation was sequenced for exon 3, SSCP analysis of exon 3, Sanger sequencing, mass array, PCR, or other methods in primary HCC tissue
4. The study with the highest quality assessment was enrolled when more than one study was reported by one individual author;
5. Studies were published in English.

Exclusion criterion:

1. Articles not related to the clinic;
2. Overlapping publications;
3. Information about *CTNNB1* mutation or OS or other clinicopathological features that were not clearly reported;
4. Abstracts, reviews, letters, editorials, and expert opinions;
5. Non-English publications.

### Data extraction and literature quality assessment

Two reviewers (GLL and GQS) independently evaluated each study, and relevant characteristics were listed: (1) the first author and publication year; (2) population origin; (3) number of cases; (4) mean age, (5) gender, (6) the number of cases with *CTNNB1* mutation; (7) level of evidence, (8) disease stage, (9) clinicopathological features, (10) methods of evaluating *CTNNB1* mutation, and (11) OS data.

The quality of each study was assessed using the Newcastle-Ottawa scale (NOS), which evaluates various aspects of the methodology, including selection, comparability, and outcome [14]. The final scores ranged from 0 (lowest) to 9 (highest); the higher the value, the better the eligibility.

### Statistical analysis

Review Manager (RevMan) software (version 5.2; Cochrane Collaboration) was used for the meta-analysis. Odds ratios (OR) combined with 95%confidence intervals (CI) were analyzed to evaluate the association of *CTNNB1* mutation with the prognosis and clinicopathological factors of HCCs. Pooled ORs and 95%CIs were used as the recommended summary statistics. A fixed- or random-effects model was used to calculate pooled effects. Funnel plots, which were used to examine the risk of potential publication bias, were constructed using Egger’s test and Begg’s test. Heterogeneity was evaluated by *I^2^*, and *I^2^* statistics of ≥50%, defined as heterogeneity. Statistical significance was set at *P <*0.05.

### Availability of data and materials

All data and materials were availability from the web.

### Consent to publication

All co-authors consented to publish this paper.

## RESULTS

### Selection of trials

**Figure 1:**
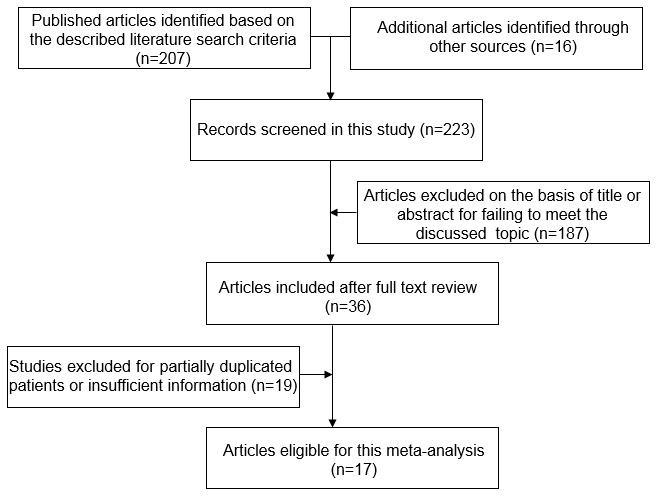
Flow chart of literature search strategies.

The original search strategy retrieved 223 publications. After screening, 187 studies were excluded, and 36 papers were captured. Of these, 19 were excluded because of a lack of adequate data on *CTNNB1* mutations and specific parameters. Thus, 17 studies with sufficient evaluation met the inclusion criteria and were retrieved for further evaluation. A flowchart of the strategies is presented in Figure 1.

### Study characteristics

The patient characteristics in each selected study are shown in Table 1. The total number of patients was 1828, with 319 *CTNNB1* mutations. The mean incidence of *CTNNB1* mutation was 17.5%. The information extracted from the selected studies included the gene type of *CTNNB1*, prognosis, disease stage, methods of evaluating *CTNNB1* mutation, OS data, and clinicopathological features. The ORs and 95% CI between *CTNNB1* mutation and OS are provided. All studies retrieved in this meta-analysis were performed properly, and the gene type of *CTNNB1* was determined by sequencing of exon 3, SSCP analysis of exon 3, Sanger sequencing, mass array, PCR, or other methods without subjective interference. The primary mutation was found in exon 3.

**Table 1:** Characteristics of studies included in the meta-analysis.

**First author and year**	**Country /region**	**No. of patients**	**Mean age**	**Gender (M/F)**	**Mu/total**	**Level of evidence**	**Stage**	**Clinicopathological features**	**Method**	**ProvidedOS data**
Ding [15] 2014	China	156	53.09±11.19	NR	15/156	5	I–IV	D,T	Mass array	Yes
Lin [16] 2010	Taiwan	160	57(14-88)	122/38	22/128	5	NR	NR	Direct sequencing of exon 3	Yes
Lu [17] 2014	Taiwan	115	56.3(23.6–83.1)	97/18	21/115	5	I–IV	T,M	Direct sequencing of exon 3	Yes
Mao [18] 2001	Taiwan	372	NR	293/162	36/372	5	I–III	D,T	PCR	Yes
Wong [19] 2001	Hong Kong	60	54(28-74)	46/14	6/60	4	I–IV	D	PCR	NR
Yuan [20] 2013	Taiwan	305	55.09 (15–88)	239/66	32/214	4	I–IV	T	direct sequencing of exon 3	Yes
Cavard [21] 2006	France	42	NR	NR	21/42	3	NR	NR	sequencing	NR
Cieply [22] 2009	USA	25	NR	19/6	9/25	3	I–IV	T,M	Direct sequencing of exon 3	NR
Hsu [23] 2000	Japan	125	63(16-79)	88/37	57/434	3	I–IV	D,T	PCR	Yes
Huang [24] 1999	Japan/ Switzerland	16+6	NR	NR	9/22	3	NR	D	DNA sequence	NR
Kim [25] 2008	Korea	36	57.7(34-71)	32/4	1/36	3	I–IV	D,T,M	sequencing	NR
Puig [26] 2001	France	137	NR	110/27	32/137	3	NR	D,M	Direct sequencing	NR
Li [27] 2011	United States	7	56.86	¾	5/7	3	I–III	T	Sanger sequencing	NR
	China	1	68	1/0	1/1					
	The Netherlands	1	53	1/0	0/1					
Park [28] 2005	Korea	92	51.6(26-89)	75/17	13/32	3	I–IV	D,T	SSCP analysis of exon 3	NR
Taniguchi [29] 2002	USA	73	NR	41/32	14/73	3	NR	D	PCR	NR
Tornesello [30] 2013	Italian	67	NR	53/14	10/67	3	NR	D	DNA sequence electropherograms	NR
Rossi [31] 2007	France	32	NR	NR	15/32	3	NR	NR	sequencing	NR

D, histologic differentiation degree; T, depth of tumor invasion; M, metastasis (include those with more than one Nodules); OS, overall survival; NR, not reported; M/F, male/female; Mu, *CTNNB1* mutation.

### Quality assessment

The methodological quality of the 17 studies was assessed using NOS. On the basis of the NOS, 4 studies scored 5 points [15–18], 2 studies scored 4 [19, 20], 11 studies scored 3 [21–31] in total of the 17 studies. Studies with a score ≥ 5 were defined as high-quality (Table 1).

### Impact of *CTNNB1* mutation on overall survival (OS)

**Figure 2:**
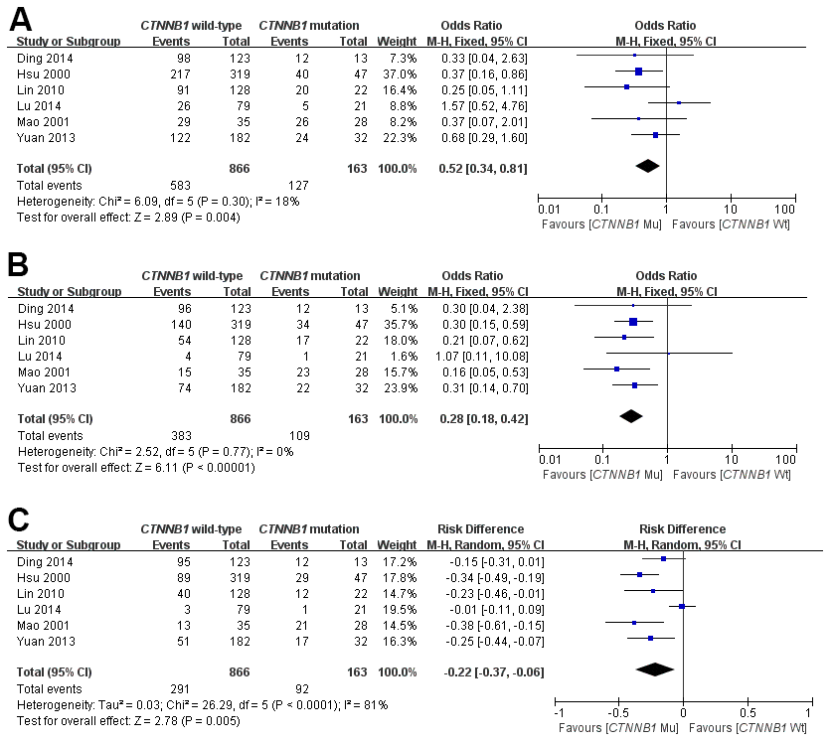
Forest plot of odds ratio for the association of *CTNNB1* mutation with 1-year (**A**), 3-year (**B**) and 5-year (**C**) overall survival.

Some of the included studies [15–18, 20, 23] provided the ORs and 95%CI directly or indirectly when discussing the correlation between *CTNNB1* mutation and *OS*. This meta-analysis systematically assessed the association of *CTNNB1* mutation with OS in patients with HCC at 1-year, 3-year and 5-year, respectively. It was demonstrated that *CTNNB1* mutation significantly correlated with poor 1-, 3-and 5-year OS, as shown in Figure 2. And the pooled ORs were 0.52(n = 6 studies, 95% CI: 0.34–0.81, Z = 2.89, *P* =0.004, 0.28 (n =6 studies, 95% CI: 0.18–0.42, Z = 6.03, *P<*0.00001), -0.22(n = 6 studies, 95% CI: 0.37–0.06, Z = 2.78, *P* = 0.005) respectively. The statistical heterogeneity of the 1-, 3-and 5-year OS was 18%, 0%, and 81%, respectively. The above results suggest that *CTNNB1* mutation is correlated with a favorable prognosis for HCC.

### Correlation of *CTNNB1* mutation with clinicopathological parameters

**Figure 3:**
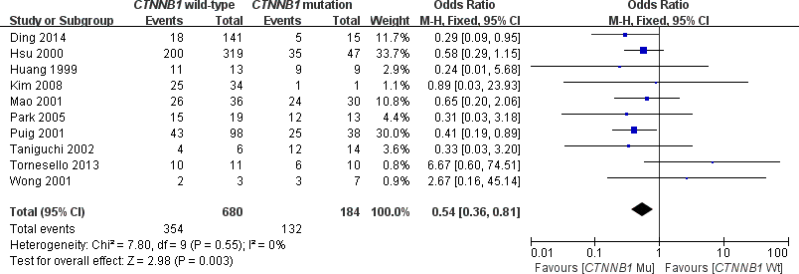
Forest plot of odds ratio for the association of *CTNNB1* mutation with differentiation grade.

**Figure 4:**
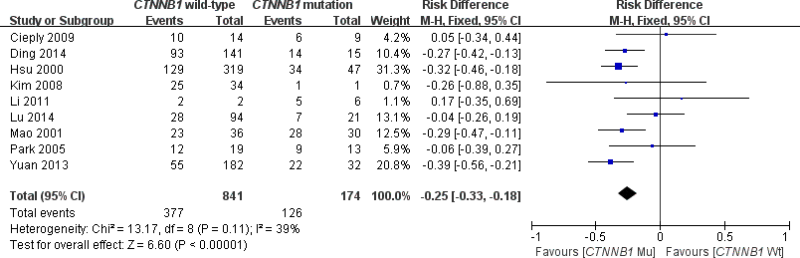
Forest plot of odds ratio for the association of *CTNNB1* mutation with TNM stages.

**Figure 5:**
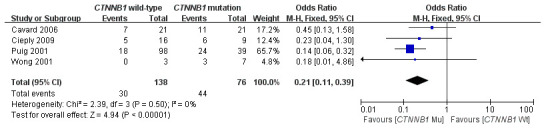
Forest plot of odds ratio for the association of *CTNNB1* mutation with liver cirrhosis.

This meta-analysis assessed the relationship between *CTNNB1* mutations and clinicopathological parameters, including metastasis, vascular invasion, tumor size, differentiation, TNM stages, liver cirrhosis, and HBV/HCV infection (Table 2). Ten studies [15, 18, 19, 23–26, 28–30] evaluated the correlation between *CTNNB1* mutations and differentiation (Figure 3). The pooled OR was 0.54 (95%CI: 0.36-0.81, Z=2.98, *P*=0.003). This result showed that there was a significant correlation between *CTNNB1* mutation and differentiation. Nine studies [15, 17, 18, 20, 22, 23, 25, 27, 28] evaluated the correlation of *CTNNB1* mutations with TNM stages (T3/T4 versus T1/T2) (Figure 4). The pooled OR was -0.25 (95%CI: -0.33--0.18, Z=6.6, *P*<0.00001). This result indicated that there was a significant correlation between *CTNNB1* mutations and the TNM stages of HCC. Additionally, we also assessed the relationship between *CTNNB1* mutation and liver cirrhosis of HCC.4 studies [19, 21, 22, 26] evaluated the correlation of *CTNNB1* mutation with liver cirrhosis (Figure 5). The pooled OR was 0.21 (95%CI: 0.11-0.39, Z=4.94, *P*<0.00001). This result showed a significant correlation between *CTNNB1* mutations and liver cirrhosis. We found that *CTNNB1* mutation had a better effect on the above two clinicopathological features. For other parameters, such as metastasis (Supplementary Figure 1), vascular invasion (Supplementary Figure 2), and tumor size (Supplementary Figure 3), of HCC showing *CTNNB1* mutation, the pooled ORs were1.25(n=4studies, 95%CI:0.93-1.66, Z=1.49, *P*=0.14),1.42 (n=3 studies, 95%CI:0.82-2.45, Z=1.26, *P*=0.21) and 1.24 (n=5 studies, 95%CI:0.37-4.11, Z=0.35, *P*=0.72), respectively, demonstrating that *CTNNB1* mutation had no significant correlation with these parameters.

**Table 2:** Meta-analysis comparing HCC with *CTNNB1* mutation and wild-type.

**Outcome of interest**	**No. of studies**	**Number of tissue samples**	**OR/WMD**	**95% CI**	**P value**	**I^2^(%)**
Overall Survival [15–18, 20, 23]						
1 year	6	*CTNNB1* Mu=163, *CTNNB1* Wt =866	0.52	0.34-0.81	0.004	18
3 year	6	*CTNNB1* Mu=163, *CTNNB1* Wt =866	0.28	0.18-0.42	<0.00001	0
5 year	6	*CTNNB1* Mu=163, *CTNNB1* Wt =866	-0.22	-0.37--0.06	0.005	81
Differentiation grade [15, 18, 19, 23–26, 28–30,]	10	*CTNNB1* Mu=184, *CTNNB1* Wt =680	0.54	0.36-0.81	0.003	0
TMN stage [15, 17, 18, 20, 22, 23, 25, 27, 28]	9	*CTNNB1* Mu=174, *CTNNB1* Wt =841	-0.25	-0.33--0.18	<0.00001	39
Metastasis [17, 22, 25, 26]	4	*CTNNB1* Mu=69, *CTNNB1* Wt =242	1.25	0.93-1.66	0.14	0
Vascular invasion [17, 22, 26]	3	*CTNNB1* Mu=70, *CTNNB1* Wt =223	1.42	0.82-2.45	0.21	43
Liver cirrhosis [19, 21, 22, 26]	4	*CTNNB1* Mu=76, *CTNNB1* Wt =138	0.21	0.11-0.39	<0.00001	0
Tumor size [19, 22, 25, 26, 28]	5	*CTNNB1* Mu=67, *CTNNB1* Wt =171	1.24	0.37-4.11	0.72	54
HBV [15, 17–19, 21, 23, 25–28, 30, 31]	12	*CTNNB1* Mu=213, *CTNNB1* Wt =795	0.44	0.31-0.64	<0.0001	0
HCV [15, 17, 21, 22, 25, 27, 28, 30, 31]	9	*CTNNB1* Mu=111, *CTNNB1* Wt =356	1.70	0.93-3.11	0.09	0

OR, odds ratio; WMD, weighted mean difference; CI, confidence interval; Mu, mutation; Wt, wild-type.

### Correlation of *CTNNB1* mutation with etiology

**Figure 6:**
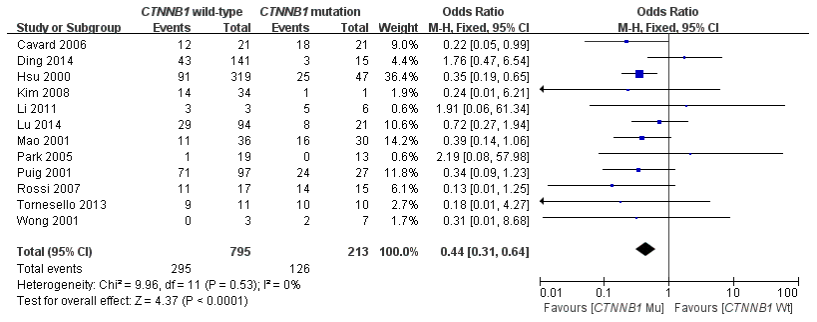
Forest plot of odds ratio for the association of *CTNNB1* mutation with etiology (HBV).

In this meta-analysis, the correlation between *CTNNB1* mutations and etiology (HBV/HCV infection) was evaluated. As shown in Figure 6, 12 studies [15, 17, 18, 19, 21, 23, 25–28, 30, 31] assessed the relationship between *CTNNB1* mutations and HBV. The combined ORs were 0.44(95%CI:0.31-0.64, Z=4.37, *P<*0.0001), with no significant statistical heterogeneity (*I*^2^=0%). This result indicates that a better effect was observed between *CTNNB1* mutation and HBV infection. However, as shown in Supplementary Figure 4, 9 studies [15, 17, 21, 22, 25, 27, 28, 30, 31] assessed the relationship between *CTNNB1* mutations and HCV. The combined ORs were 1.70(95%CI: 0.93-3.11, Z=1.72, *P*=0.09).

### Publication bias

For the studies included in this meta-analysis, Begg’s test indicated that there was no significant publication bias after assessing the funnel plot (Supplementary Figures 5–15).

## DISCUSSION

Human HCCs with activation of the Wnt/β-catenin pathway demonstrate unique gene expression patterns and pathological features. Activated Wnt/β-catenin synergizes with multiple signaling cascades to drive HCC formation, and it functions through its downstream effectors [32]. The aberrant Wnt pathway in HCC, has been well-studied and proven to be involved in the prognosis of HCC [33]. β-catenin is a key downstream effector of the Wnt signaling pathway and plays a crucial role. β-catenin has been the focus of attention as an attractive therapeutic target [9]. According to our previous observations, cytoplasmic and/or nuclear expression of β-catenin could serve as a potential predictor for the progression and prognosis of patients with HCC and act as a novel target for the developed therapies [13]. *CTNNB1*, the coding gene of β-catenin, is one of the most frequently mutated genes in HCC [10, 34]. Hsu et al. first reported a relationship between β-catenin mutations and prognosis in patients with HCC [23]. Based on the resected HCC of patients, their report showed that *CTNNB1* mutations were associated with tumor differentiation, HBV infection, and clinical prognosis. However, according to a study by Lu et al., there is no prognostic significance of *CTNNB1* mutation in patients with HCC [17]. Moreover, no difference in *CTNNB1* mutation rate was observed between patients with HCC with or without HBV infection. Tumor mutational burden (TMB) was verified to be closely associated with immune checkpoint inhibitors, but it is unclear whether gene mutation has an effect on immunotherapy of HCC. Mo et al. firstly revealed the underlying association between *CTNNB1* mutation and immunotherapy, and they speculated that *CTNNB1* mutation may modulate NK cells by affecting CD96 [35]. To date, however, tremendous work has been done to investigate the association between *CTNNB1* mutations and clinicopathological characteristics and prognosis of patients with HCC; however, no conclusive results have been achieved. Wang et al. reported that the *CTNNB1* mutation was correlated with a favorable prognosis in HCC in a meta-analysis [36]. Of note, the studies enrolled in their meta-analysis failed to provide adequate information about the relationship between *CTNNB1* mutations and clinical prognosis. The current study focused on *CTNNB1* mutations in HCC in a clinical setting. We showed that *CTNNB1* mutation has a robust effect on the clinical and prognosis of patients with HCC.

A number of studies have implicated an alteration in *CTNNB1*, including gene mutation and protein overexpression, in HCC [34, 37]. *CTNNB1* plays a crucial role in hepatocyte adhesion and Wnt signaling pathway [38]. *CTNNB1* is mainly located in the cell membrane. By binding to the lymphoid enhancer factor (LEF)/T-cell factor (TCF) family of DNA binding proteins, *CTNNB1* enters the nucleus and regulates transcription of target genes such as c-*myc*or cyclin D1, resulting in proliferation and metastasis of liver tumor cells [39]. Xiao X et al. demonstrated that *CTNNB1* mutation in HCC led to a decrease in chemokine expression and subsequent suppression of immune cell infiltration [40]. Recently, somatic mutations in *CTNNB1* have been demonstrated not only in animal models but also in human HCC [10]. Comprehensive analysis of clinical samples has identified immunological and molecular classification of HCC, and the *CTNNB1*-mutated subtype exhibits distinctive characteristics of immunosuppressive tumor microenvironment [41]. However, the clinical implications of *CTNNB1* mutation in human HCC are unclear [12, 42]. Paradoxical data exist concerning the prognostic value and clinicopathological significance of cytoplasmic and/or nuclear *CTNNB1* accumulation. These discrepancies are most likely due to the gene type of *CTNNB1*. Cytoplasm and/or nucleus*CTNNB1* accumulation can be associated with mutations in the *CTNNB1* gene and other components of the signaling pathway [43]. The most common mechanism of *CTNNB1* accumulation in HCC is mutations in *CTNNB1* [44]. Mutations or wild-type *CTNNB1* may influence the subcellular localization of β-catenin. Most studies on *CTNNB1* mutations have focused on the consensus sequence for GSK-3β phosphorylation in exon 3 and the inactivation of APC and other factors [45]. Cui et al. found that mutation of exon3 of *CTNNB1* is one of the most important factors activating the abnormal Wnt signaling pathway in HCC [46]. *CTNNB1* mutations and nuclear overexpression may play a key role in HCC in Chinese people. And targeting the Wnt-beta-catenin pathway may represent a valid treatment option for Chinese HCC patients [15, 47, 48].

In the past 5 years, immune-checkpoint inhibitors have revolutionized the management of HCC [49]. However, several studies demonstrated that mutation of *CTNNB1* have also been associated with scarcity of immune cells in the tumor microenvironment and poor clinical response to immune checkpoint inhibitor therapy [50]. Ogawa K et al. verified that Gain-of-function Inclusion mutation of *CTNNB1* contributes to resistance of ICI monotherapy through the framework of non-T-cell-inflamed tumor microenvironment. Of note, the treatment effect of Atezolizumab plus bevacizumab in patients with HCC with MT *CTNNB1* was comparable to those patients with WT *CTNNB1*. These results further implicate that bevacizumab added to Atezolizumab might improve immunosuppressive tumor microenvironment caused by *CTNNB1* mutation [51]. In addition, Chen et al. proved that *CTNNB1* alternation is a potential biomarker for immunotherapy prognosis in patients with HCC [52]. However, this meta-analysis concluded that HCC patients with *CTNNB1* mutations appeared to have a favorable survival in comparison with wild-type *CTNNB1* HCC. Additionally, *CTNNB1* mutations were significantly associated with the differentiation grade, TNM stage, liver cirrhosis, and HBV infection. The reason may be closely related to the concept of *CTNNB1* mutations in this meta-analysis refer to loss-of-function mutation.

However, our study has some limitations. Firstly, the clinical data used in this study were acquired from a relatively small cohort in each study, so selection bias or potential biases related to imbalanced clinical characteristics is inevitable. This will also lead to heterogeneity, which is a potential problem that may affect the results of all meta-analyses. In this study, significant heterogeneity was found when discussing the relationship between *CTNNB1* mutations and 5-year overall survival and tumor size in the selected studies. Unfortunately, due to limited information, a meta-regression analysis could not be conducted. To eliminate variations across the included studies, we used a random effects model. Although this method may not completely eliminate the effects of heterogeneity, its adverse effects must be weakened. The second was publication bias, which can be seen in the publication bias evaluation. As is well known, this bias was unavoidable because positive results were more likely to be published than negative ones. Moreover, most studies included in this meta-analysis failed to elucidate the relationship between *CTNNB1* expression and *CTNNB1* mutation. Some studies provided the number of tissue samples with *CTNNB1* expression, but failed to provide information on the gene type of *CTNNB1*, and vice versa. What’s more, the method of detecting *CTNNB1* mutations was not standardized from author to author. Direct sequencing of exon 3, mass array, DNA sequence electropherograms, Sanger sequencing, SSCP analysis of exon 3, and PCR were performed. Therefore, a more standardized analysis should be performed and more prospective works and experimental clinical research should be conducted. Last but not the least, how *CTNNB1* mutation effect clinical outcomes and survival of patients with HCC and how it serves as a valuable prognostic predictor is indeterminacy. Further research on the regulation of *CTNNB1* expression by the *CTNNB1* mutation-related signaling pathway is needed and will help to elucidate the new mechanism of drug resistance, providing a theoretical basis for the prediction of drug sensitivity in HCC and the development and application of new therapeutic targets for reversing drug resistance.

In conclusion, *CTNNB1* mutation could serve as a potential predictor for the clinical and prognosis of HCC patients and act as a novel useful biomarker of molecular targeted therapies for HCC.

## Supplementary Material

Supplementary Figures
