# Supplementary material for: Prognostic significance of CTNNB1 mutation in hepatocellular carcinoma: a systematic review and meta-analysis
Source: Aging (Albany NY). 2023 Sep 20;15(18):9759–78. doi: 10.18632/aging.205047 (PMC10564414; doi:10.18632/aging.205047)
Supplement: Supplementary Figures [file aging-15-205047-s001.pdf]

SUPPLEMENTARY FIGURES

Study or Subgroup	*CTNNB1* wild-type Events	*CTNNB1* wild-type Total	*CTNNB1* mutation Events	*CTNNB1* mutation Total	Weight	Risk Ratio M-H, Fixed, 95% CI
Cieply 2009	10	16	6	9	17.1%	0.94 [0.52, 1.70]
Kim 2008	29	34	1	1	6.3%	1.12 [0.50, 2.53]
Lu 2014	28	94	6	21	21.9%	1.04 [0.50, 2.19]
Puig 2001	63	98	17	38	54.7%	1.44 [0.98, 2.11]
**Total (95% CI)**		**242**		**69**	**100.0%**	**1.25 [0.93, 1.66]**
Total events	130		30			

Heterogeneity: Chi² = 1.68, df = 3 (P = 0.64); I² = 0%
Test for overall effect: Z = 1.49 (P = 0.14)

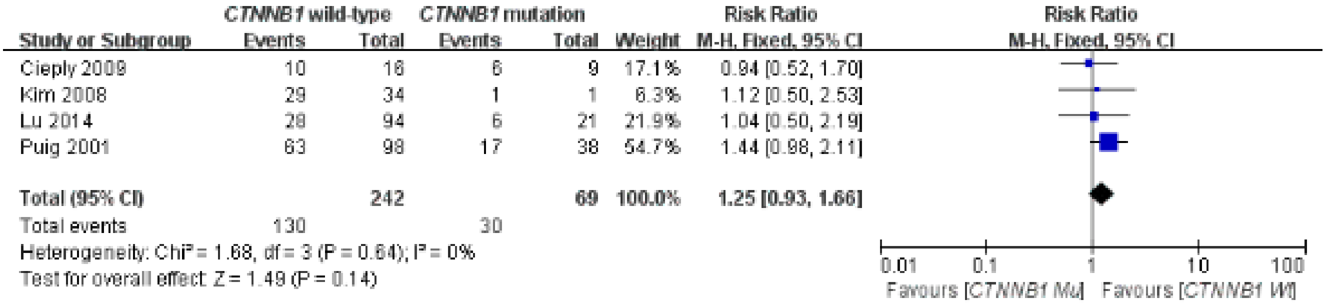

Supplementary Figure 1. Forest plot of odds ratio for the association of *CTNNB1* mutation with metastasis.

Study or Subgroup	*CTNNB1* wild-type Events	*CTNNB1* wild-type Total	*CTNNB1* mutation Events	*CTNNB1* mutation Total	Weight	Odds Ratio M-H, Fixed, 95% CI
Cieply 2009	10	16	2	9	4.4%	5.83 [0.90, 37.82]
Lu 2014	55	115	11	21	45.0%	0.83 [0.33, 2.11]
Puig 2001	56	92	20	40	50.6%	1.56 [0.74, 3.29]
**Total (95% CI)**		**223**		**70**	**100.0%**	**1.42 [0.82, 2.45]**
Total events	121		33			

Heterogeneity: Chi² = 3.51, df = 2 (P = 0.17); I² = 43%
Test for overall effect: Z = 1.26 (P = 0.21)

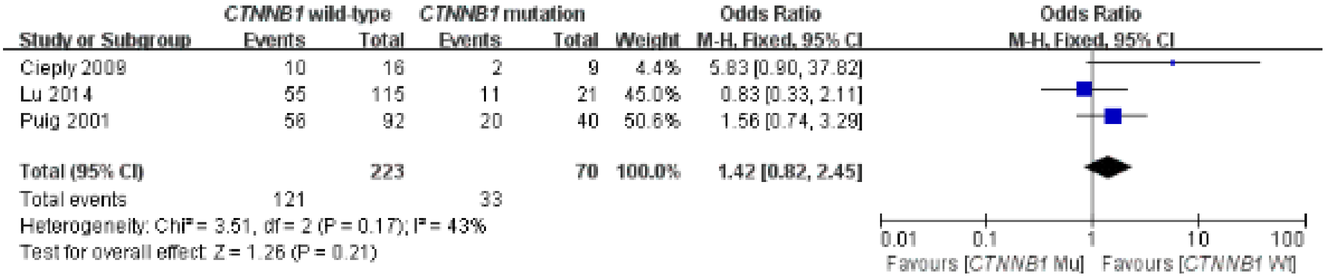

Supplementary Figure 2. Forest plot of odds ratio for the association of *CTNNB1* mutation with vascular invasion.

Study or Subgroup	*CTNNB1* wild-type Events	*CTNNB1* wild-type Total	*CTNNB1* mutation Events	*CTNNB1* mutation Total	Weight	Odds Ratio M-H, Random, 95% CI
Cieply 2009	13	16	5	9	21.2%	3.47 [0.56, 21.35]
Kim 2008	28	34	1	1	9.9%	1.46 [0.05, 40.11]
Park 2005	9	19	10	13	24.1%	0.27 [0.06, 1.30]
Puig 2001	56	99	12	38	35.0%	2.82 [1.28, 6.22]
Wong 2001	0	3	2	6	9.8%	0.26 [0.01, 7.27]
**Total (95% CI)**		**171**		**67**	**100.0%**	**1.24 [0.37, 4.11]**
Total events	106		30			

Heterogeneity: Tau² = 0.90; Chi² = 8.68, df = 4 (P = 0.07); I² = 54%
Test for overall effect: Z = 0.35 (P = 0.72)

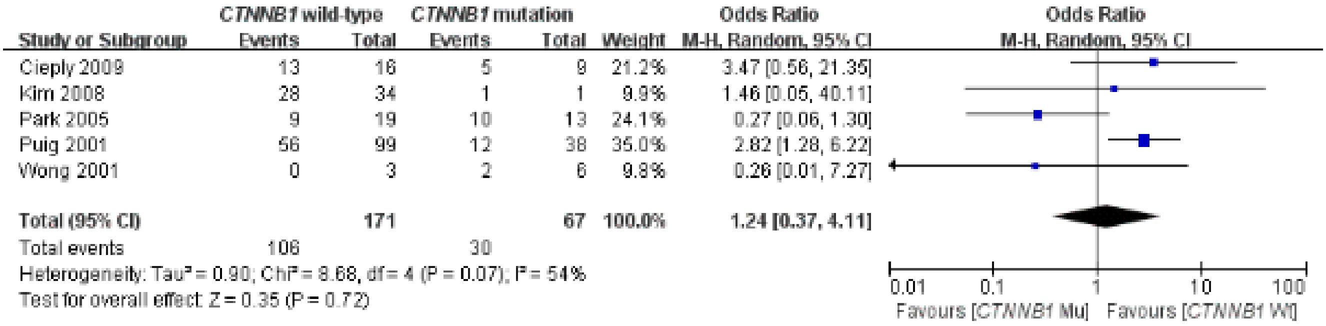

Supplementary Figure 3. Forest plot of odds ratio for the association of *CTNNB1* mutation with tumor size.

	CTNNB1 wild-type		*CTNNB1* mutation			Odds Ratio
Study or Subgroup	Events	Total	Events	Total	Weight	M-H, Fixed, 95% CI
Cavard 2006	16	21	12	21	18.4%	2.40 [0.64, 9.03]
Cieply 2009	13	16	6	9	9.3%	2.17 [0.33, 14.06]
Ding 2014	140	141	15	15	1.9%	3.02 [0.12, 77.41]
Kim 2008	30	34	1	1	2.3%	2.26 [0.08, 64.40]
Li 2011	1	3	2	6	5.7%	1.00 [0.05, 18.91]
Lu 2014	75	94	16	21	34.0%	1.23 [0.40, 3.79]
Park 2005	18	19	13	13	7.7%	0.46 [0.02, 12.10]
Rossi 2007	12	17	9	15	18.1%	1.60 [0.37, 6.95]
Tornesello 2013	2	11	0	10	2.7%	5.53 [0.23, 130.34]
**Total (95% CI)**		**356**		**111**	**100.0%**	**1.70 [0.93, 3.11]**
Total events	307		74			

Heterogeneity: Chi² = 2.07, df = 8 (P = 0.98); I² = 0%

Test for overall effect: Z = 1.72 (P = 0.09)

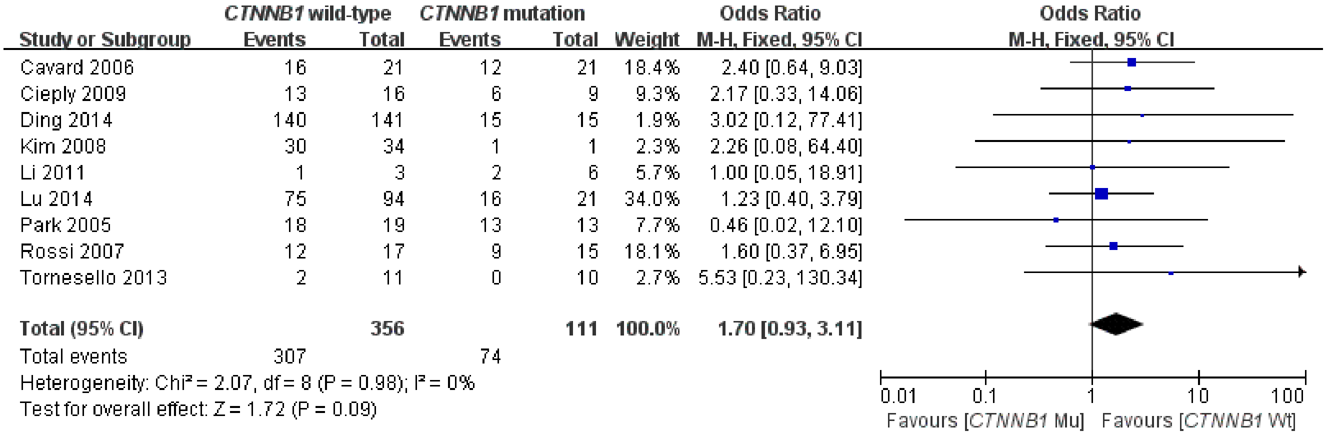

Supplementary Figure 4. Forest plot of odds ratio for the association of *CTNNB1* mutation with etiology (HCV).

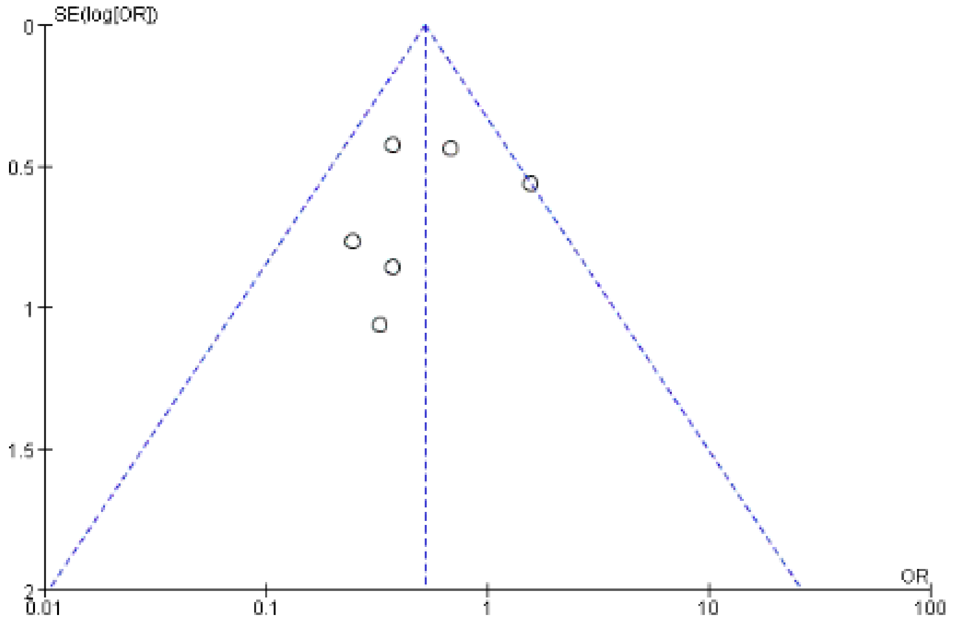

Supplementary Figure 5. Funnel plot to assess publication bias. Begg's publication bias plot showed no publication bias for studies regarding *CTNNB1* mutation and 1-year overall survival (OS) in the meta-analysis.

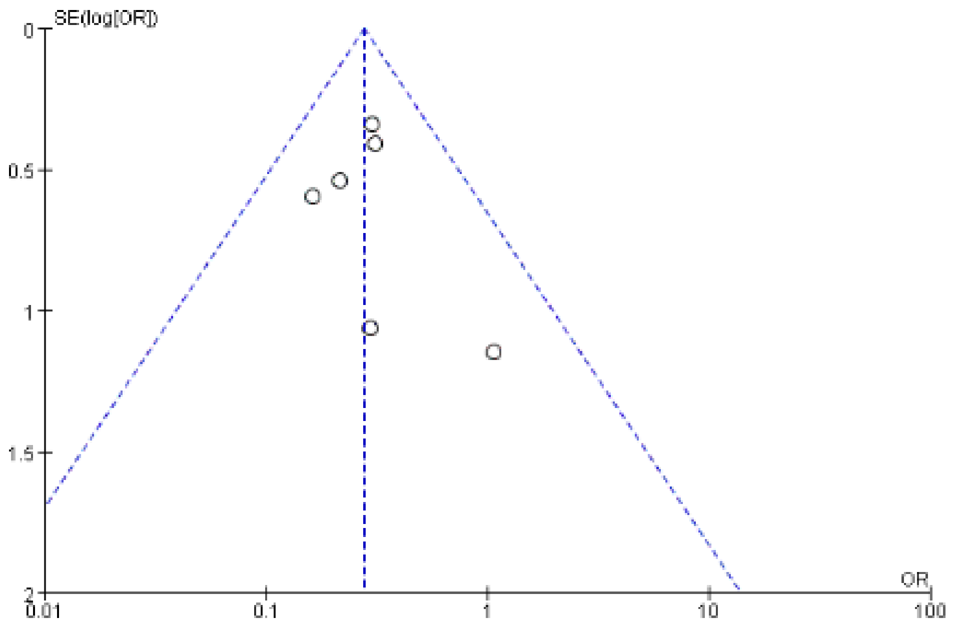

Supplementary Figure 6. Funnel plot to assess publication bias. Begg's publication bias plot showed no publication bias for studies regarding *CTNNB1* mutation and 3-year overall survival (OS) in the meta-analysis.

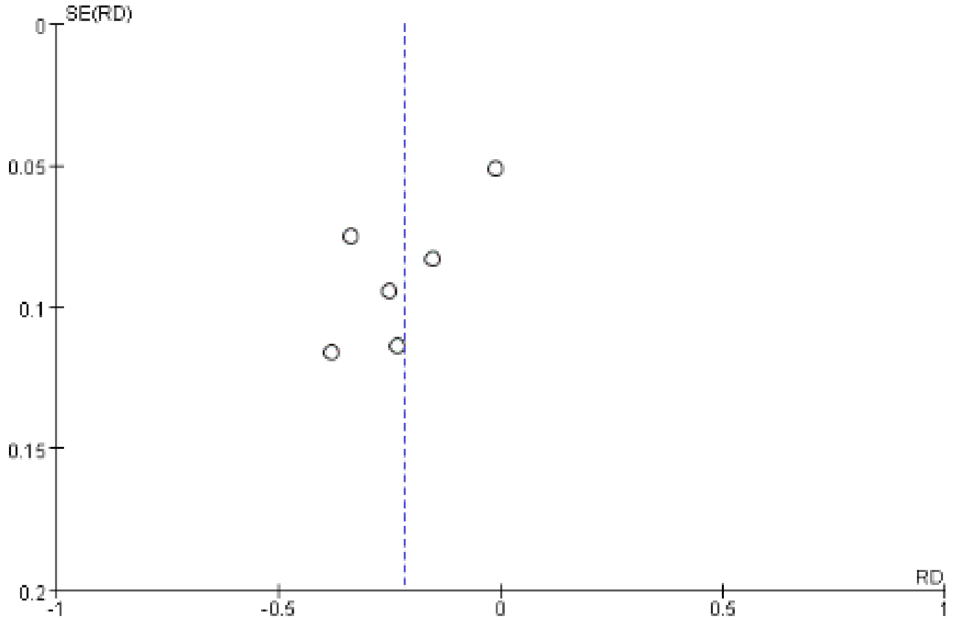

Supplementary Figure 7. Funnel plot to assess publication bias. Begg's publication bias plot showed no publication bias for studies regarding *CTNNB1* mutation and 5-year overall survival (OS) in the meta-analysis.

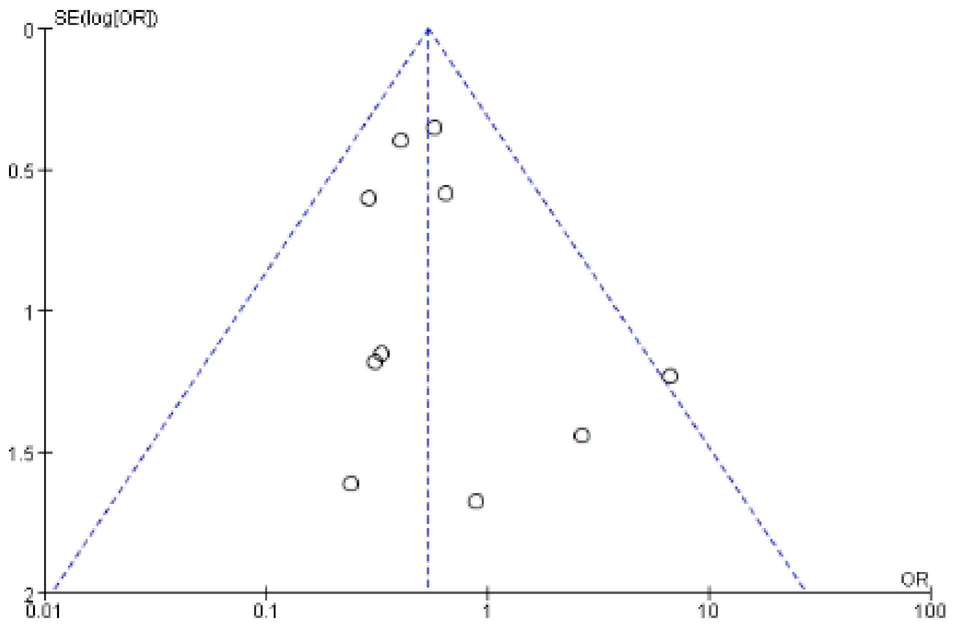

Supplementary Figure 8. Funnel plot to assess publication bias. Begg's publication bias plot showed no publication bias for studies regarding *CTNNB1* mutation and differentiation grade in the meta-analysis.

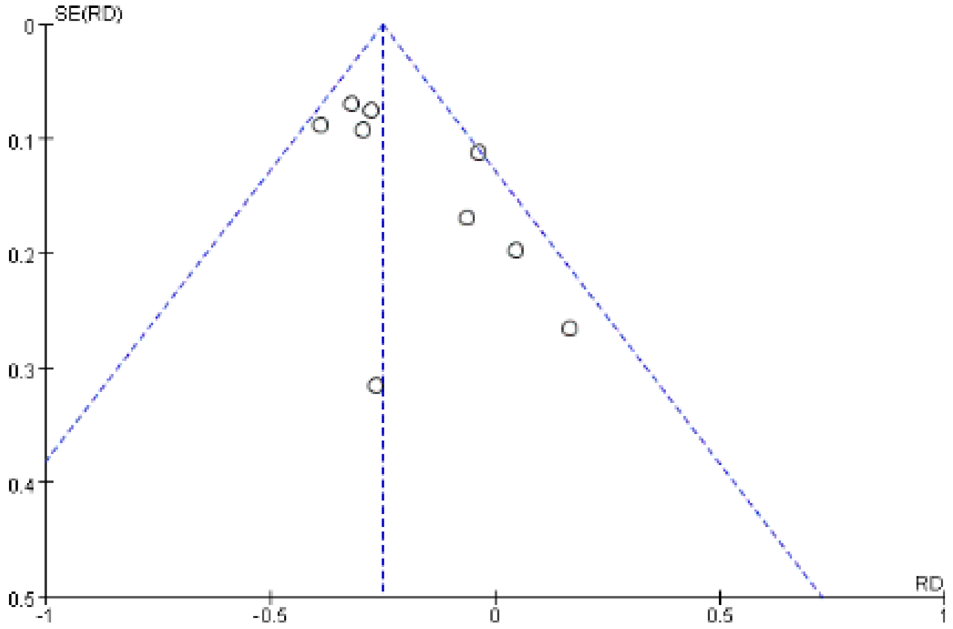

Supplementary Figure 9. Funnel plot to assess publication bias. Begg's publication bias plot showed no publication bias for studies regarding *CTNNB1* mutation and TNM stages in the meta-analysis.

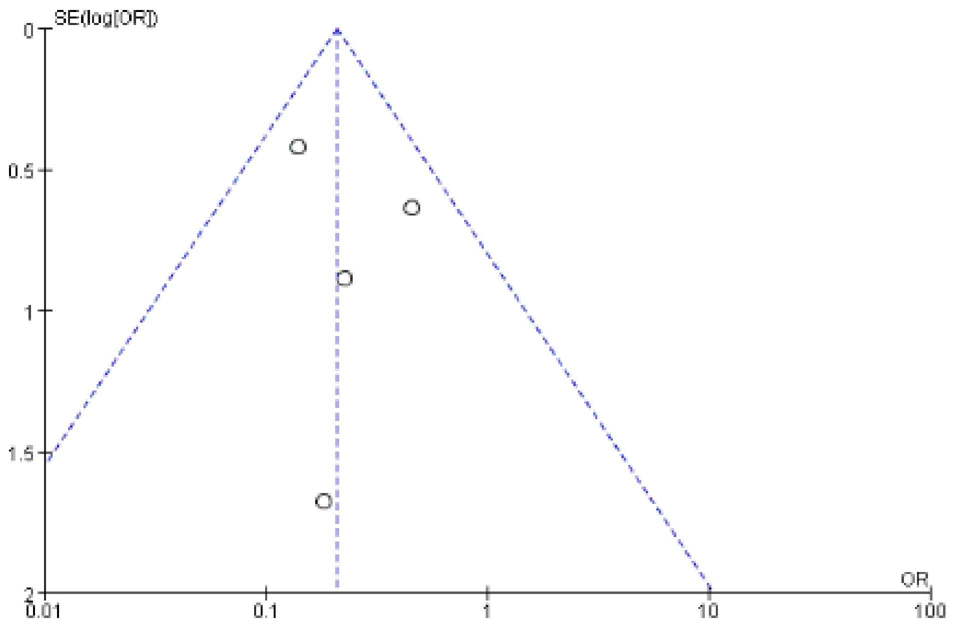

Supplementary Figure 10. Funnel plot to assess publication bias. Begg's publication bias plot showed no publication bias for studies regarding *CTNNB1* mutation and liver cirrhosis in the meta-analysis.

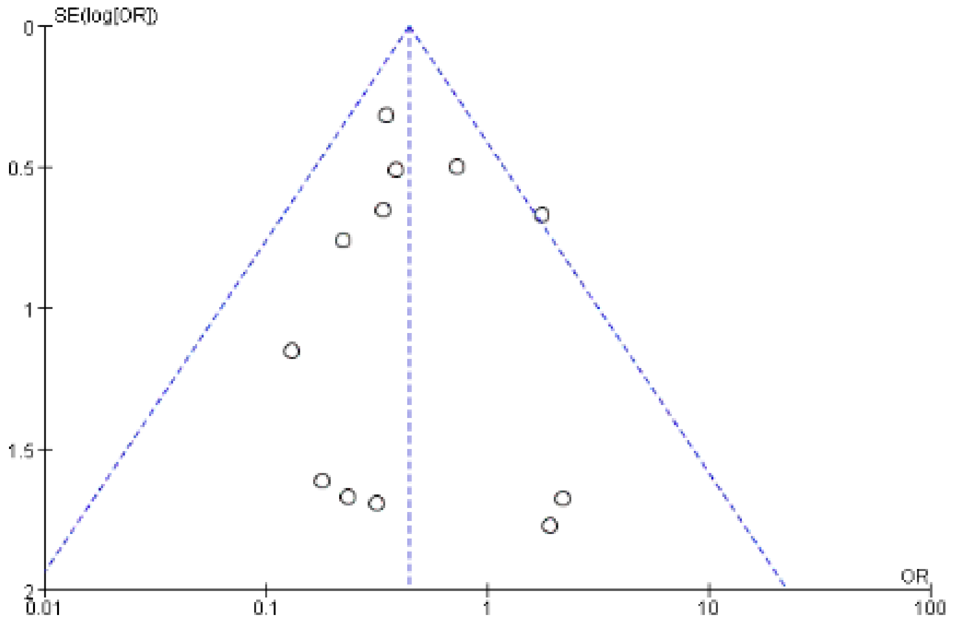

Supplementary Figure 11. Funnel plot to assess publication bias. Begg's publication bias plot showed no publication bias for studies regarding *CTNNB1* mutation and etiology (HBV) in the meta-analysis.

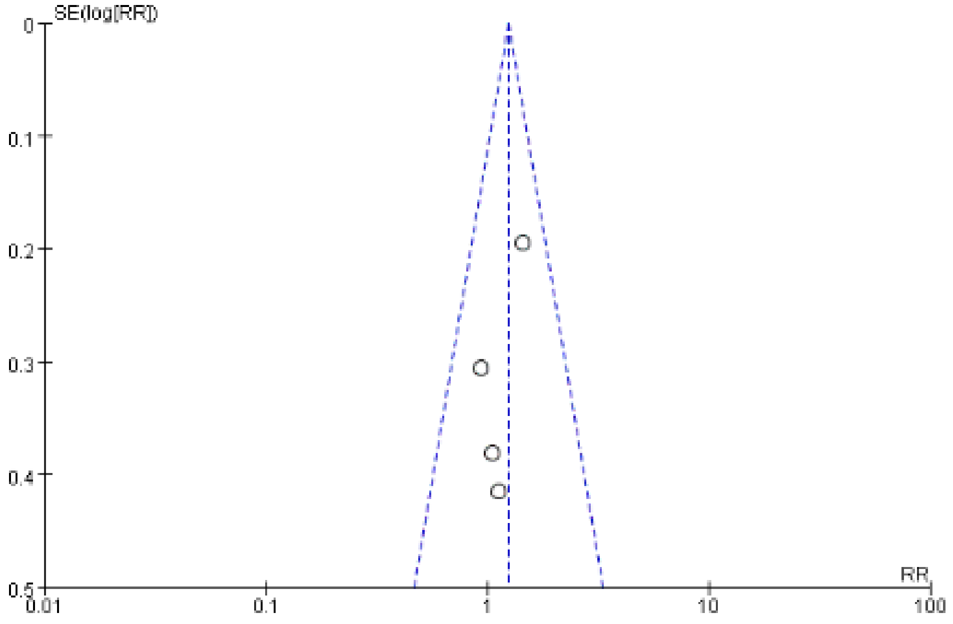

Supplementary Figure 12. Funnel plot to assess publication bias. Begg's publication bias plot showed no publication bias for studies regarding *CTNNB1* mutation and metastasis in the meta-analysis.

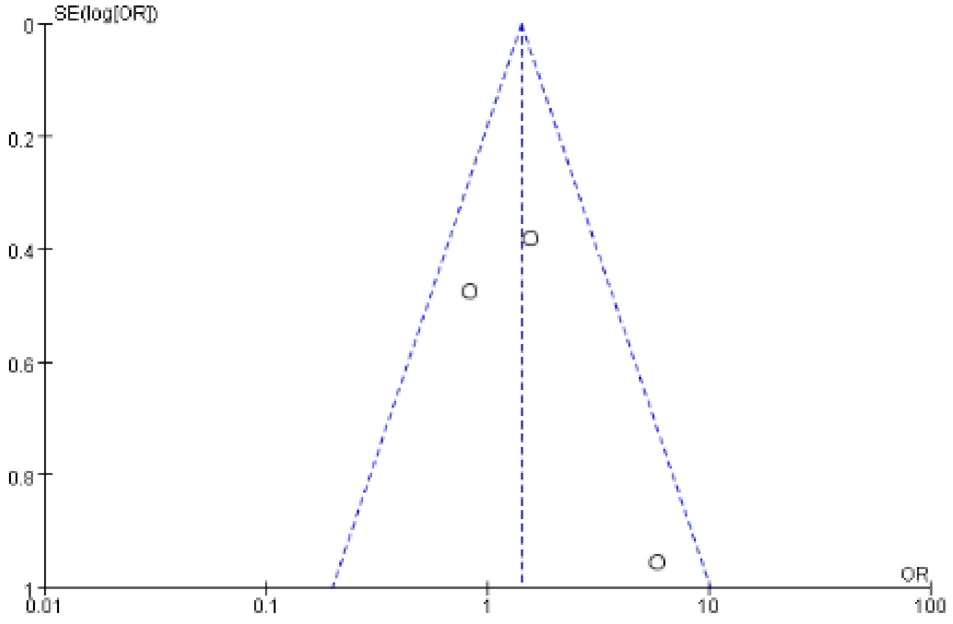

Supplementary Figure 13. Funnel plot to assess publication bias. Begg's publication bias plot showed no publication bias for studies regarding *CTNNB1* mutation and vascular invasion in the meta-analysis.

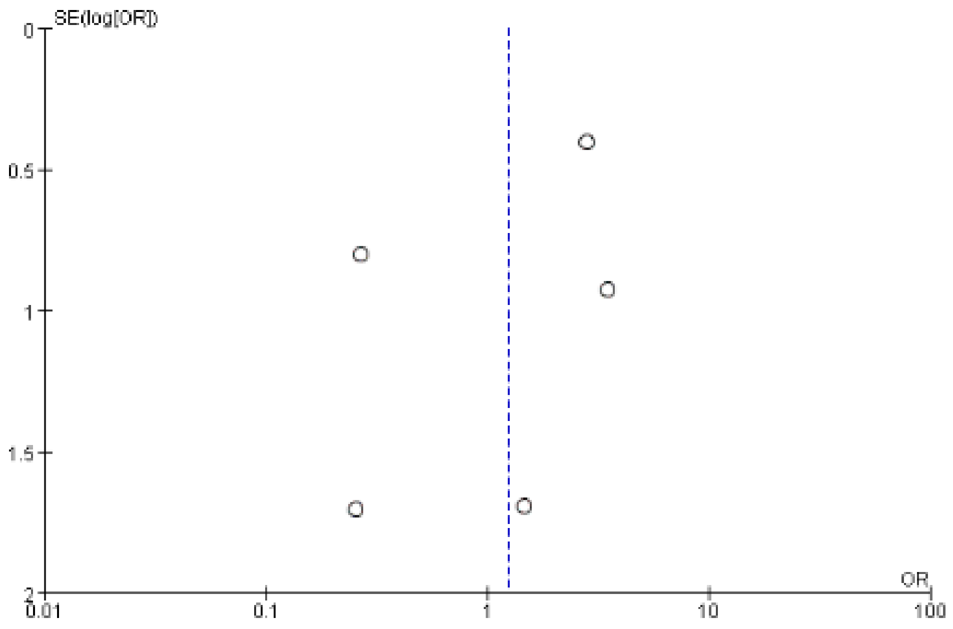

Supplementary Figure 14. Funnel plot to assess publication bias. Begg's publication bias plot showed no publication bias for studies regarding *CTNNB1* mutation and tumor size in the meta-analysis.

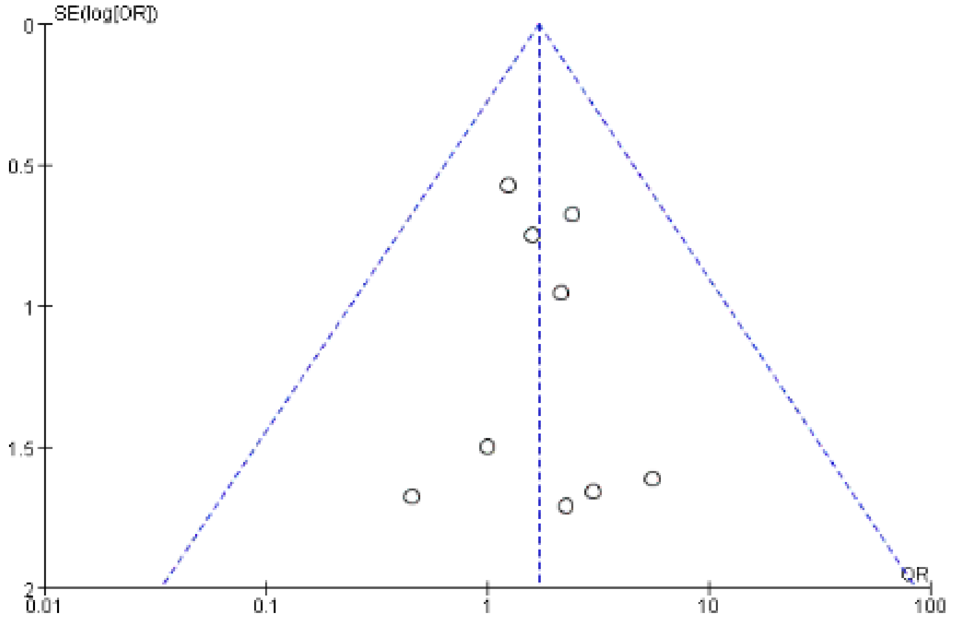

Supplementary Figure 15. Funnel plot to assess publication bias. Begg's publication bias plot showed no publication bias for studies regarding *CTNNB1* mutation and HCV in the meta-analysis.
